# Evaluation of the efficacy of Myco/F lytic system, MGIT960 system and Lowenstein-Jensen medium for recovery of *Mycobacterium tuberculosis* from sterile body fluids

**DOI:** 10.1038/srep37757

**Published:** 2016-11-23

**Authors:** Guirong Wang, Xinting Yang, Junping Zhu, Weijie Dong, Mailing Huang, Guanglu Jiang, Liping Zhao, Sibing Qin, Xiaoyou Chen, Hairong Huang

**Affiliations:** 1 National Clinical Laboratory on Tuberculosis, Beijing Key Laboratory for Drug Resistant Tuberculosis Research, Beijing Chest Hospital, Capital Medical University, Beijing Tuberculosis and Thoracic Tumor Institute, Beijing, China; 2 Department of Tuberculosis, Beijing Chest Hospital, Capital Medical University, Beijing Tuberculosis and Thoracic Tumor Institute, Beijing, China; 3 Department of Pathogenic Biology, School of Basic Medical Sciences, Capital Medical University, Beijing, China; 4 Department of Orthopedics, Beijing Bone and Joint Tuberculosis Diagnosis and Treatment Center, Beijing Chest Hospital, Capital Medical University, Beijing Tuberculosis and Thoracic Tumor Institute, Beijing, China

## Abstract

The diagnosis of extrapulmonary tuberculosis (EPTB) is challenging due to non-specific symptoms, invasive approach for specimen collection and most importantly, the paucibacillary status. The objective of this assay was to evaluate the efficacy of Myco/F lytic system, BACTEC Mycobacteria Growth Indicator Tube (MGIT) 960 system and Lowenstein-Jensen (L-J) medium for recovery of bacilli from sterile body fluids. 214 specimens (114 pleural fluid and 100 pus) from clinically diagnosed EPTB patients were collected and subjected to Ziehl-Neelsen (ZN) smear microscopy, L-J culture, MGIT 960 culture and Myco/F lytic culture.103 out of the 214 sterile body fluid samples yielded positive culture outcomes by any of the three methods. Among all the culture positive specimens, the recovery rate was 86.41% for Myco/F lytic, 75.73% for MGIT 960, and 42.72% for L-J medium. The mean time to positivity (TTP) was 27.06 ± 8.03 days for Myco/F lytic, 22.20 ± 7.84 days for MGIT960 and 42 ± 8.84 days for L-J medium. The rates of contamination were 6.54%, 3.74% and 2.80% for Myco/F lytic, MGIT960 and L-J medium respectively. Both Myco/F lytic and MGIT960 system were superior to L-J medium for recovery of bacilli from sterile body fluids. Myco/F lytic system was more favorable than MGIT960 regarding recovery rate and cost-effectiveness, thus can be considered as a promising alternative to MGIT960 system for diagnosing EPTB.

Tuberculosis (TB) is still a serious global public health problem and a leading cause of morbidity and mortality throughout the world. In 2014, an estimated 9.6 million incident cases of TB were reported globally, 22% of which were extrapulmonary tuberculosis (EPTB) cases^1^. EPTB mainly includes tuberculous lymphadenitis, pleural TB, osteoarticular TB, central nervous system TB, abdominal TB, genitourinary TB and tuberculous pericarditis^2^. Early diagnosis of EPTB is essential in instituting effective and timely therapy, however, its diagnosis presents challenges because of non-specific symptoms, paucibacillary clinical samples and difficulties in obtaining specimen^3^. Despite the significant advances in molecular diagnosis of TB during last two decades^4^, mycobacterial culture of the body fluid or biopsy specimens remains the gold standard for the diagnosis of EPTB, enabling complete post-culture antimicrobial susceptibility testing and genotyping^5^.

The use of the automated liquid culture system has considerably improved the recovery and decreased the detection time required for mycobacteria^6^,^7^,^8^,^9^. However, the routine practices labor-intensive i.e. the specimens are collected in clinics or wards by healthcare workers, then transported to the laboratories for further processing by the laboratory staff. Furthermore, multiple staves involved, many manipulation steps needed, all increase the risk of contamination. The Myco/F lytic system is a closed, fully automated, continuous-monitoring system which requires inoculation of blood or sterile body fluid samples directly into the medium without any other manipulation. The whole process is often conducted by the person who collected the specimen^10^. Studies have shown that Myco/F lytic system is not only rapid and simple but also safe and highly reliable diagnostic method for the detection of mycobacteria in blood^11^,^12^,^13^. Up to now, only one study has evaluated the performance of Myco/F lytic system for recovery of *M. tuberculosis* from pleural fluid, and the results showed that the Myco/F lytic system can be an alternative to the MGIT system for diagnosing pleural TB^14^.

The purpose of this study was to evaluate the efficacy of Myco/F lytic system, MGIT system and L-J medium for recovery of mycobactetia from sterile body fluids in terms of recovery rate, time to positivity, and contamination rate.

## Results

### Recovery of mycobacteria

A total of 103 specimens (48.13%) from103 EPTB patients out of the214 sterile body fluid samples were culture positive according to any of the three culture methods, and the Capilia TB-Neo assay identified them all as *M. tuberculosis* complex (MTC) isolates. The Myco/F lytic, MGIT 960 systems and L-J medium detected 86.41%, 75.73% and 43.72% of all the culture positive specimens, respectively (Table 1). Statistically significant differences were observed between liquid culture methods and L-J culture (P < 0.001for both comparisons). However, the difference between Myco/F lytic and MGIT 960 was not significant (P = 0.05). The combined recovery rates of each two different culture methods were analyzed. The recovery rate for Myco/F lytic plus L-J medium was 94.17% (97/103), was 82.52% (85/103) for MGIT 960 plus L-J medium, and was 98.06% (101/103) for Myco/F lytic plus MGIT 960. Statistically significant differences were observed between Myco/F lytic plus MGIT 960 and MGIT 960 plus L-J medium (P = 0.000), between Myco/F lytic plus L-J medium and MGIT 960 plus L-J medium (P = 0.009), but not between Myco/F lytic plus MGIT 960 and Myco/F lytic plus L-J (P = 0.149). Smear examinations were positive for acid-fast bacilli (AFB) for 34 of the 103 culture-positive specimens (33.01%) (Table 2).

### Time to positivity (TTP)

The mean (range) TTP was27.06 ± 8.03 (10.46 to 40.46) days, 22.20 ± 7.84 (7 to 40) days and 42 ± 8.84 (21 to 56) days for Myco/F lytic, MGIT960and L-J medium, respectively. Statistically significant differences were observed between Myco/F lytic and L-J medium, and also between MGIT960 and L-J medium (P < 0.001 for both). The difference between the two liquid media was also statistically significant (P = 0.002).

### Contamination

The contamination rates were 6.54% (14/214), 3.74% (8/214), and 2.80% (6/214) for Myco/F lytic, MGIT960, and L-J medium, respectively. No statistically significant differences were observed between any two different methods considering contamination rates. Four specimens were reported as contaminated by Myco/F lytic but were culture positive by at least one of the other two culture methods, while one specimen produced contaminated outcome by L-J medium but was successfully recovered by both liquid media.

## Discussion

Mycobacterial culture is the gold standard for TB diagnosis, and enabling post-culture drug susceptibility testing (DST) and genotyping. However, culture recovery rate for EPTB is significantly lower than that for pulmonary disease, leading to greater diagnostic and management uncertainty in this group. Thus, improving the recovery rate of the clinical specimens is important for EPTB patient care.

In the present study, efficacy of the Myco/F lytic system, MGIT 960 system and L-J medium for recovery of MTC from sterile body fluids were evaluated. As a single system, the Myco/F lytic system detected the greatest number of MTC, 89 isolates (86.41%), followed by the MGIT 960system, with 78 isolates (75.73%), and the L-J meidium, with 44 isolates (42.72%). Comparison of the individual performance of each method showed that both liquid media were superior to the conventional L-J medium, a result which is now well established by cohorts of liquid media assays^15^. Although the difference of recovery rate between Myco/F lytic and MGIT 960 was not significant (P = 0.05), yet additional 11 mycobacterial isolates were recovered by Myco/F lytic but not by the MGIT 960 system. Increasing the specimen number may assist in acquiring a significant difference. Notably, among all the culture positive specimens, Myco/F lytic had more obviously elevated recovery rate for smear negative specimens when compared with MGIT 960 (92.75% V.S. 78.76%, P = 0.016), but not for the smear positive specimens (70.59% V.S. 67.65%, P = 0.793) (Table 2).

Since individual medium cannot perfectly detect all of the mycobacteria-positive specimens, a combination of solid and liquid media is generally recommended for primary isolation of mycobacteria to increase the sensitivity of cultivation and accelerate diagnosis^16^. In the present study, higher recovery rates were noted when combined any two of the three culture methods. Myco/F lytic plus L-J medium had higher recovery rate than that of MGIT 960 plus L-J medium (P = 0.009). Although Myco/F lytic plus MGIT960 yielded the highest recovery rate, there was no statistic difference between this combination and Myco/F lytic plus L-J media (P = 0.149). Considering the cost, Myco/F lytic plus L-J medium seems to be an economic and efficient combination.

MGIT960 had shorter TTP in contrast with both L-J culture (22.20 ± 7.84 V.S. 42 ± 8.84 days) and Myco/F lytic culture (22.20 ± 7.84 V.S. 27.06 ± 8.03 days). This may have been caused by the different gradient of the broth of the two systems. Differences in the culture contamination rate between Myco/F lytic and MGIT960 liquid culture (6.54% versus 3.74%) was not statistically significant. The contamination rate for the MGIT960 system was similar as previously reported by Somoskovi A *et al.*^17^, but lower than the values in other studies using MGIT960 i.e., 12% by Harausz E *et al.*^14^, 8.6% by Cruciani M *et al.*^18^. The contamination rate for Myco/F lytic was lower than the only previous study evaluating the Myco/F lytic system from pleural fluid (19%)^14^. The lower contamination rate in our assay is likely a result of better aseptic operation during the specimen collection and processing, while different patient population is a reasonable cause as well^19^.

It is generally agreed that the volume of specimen can affect the yield of cultures. In this assay, pleural fluid specimens were always enough to perform all the tests. Among the 36 culture positive pleural fluid specimens, Myco/F lytic had apparent higher recovery rate (88.89%) than those of MGIT 960 (69.44%) and L-J medium (36.11%) (Table 1), which indicated that specimen volume variation was not the main cause of the enhancement. For pus specimens, no enough volume of pus could impair the recovery rates of cultures (L-J and MGIT960) that performed after Myco/F lytic assay. However, Myco/F lytic assay had less recovery rate enhancement for pus than for pleural fluid specimens compared with MGIT960 and L-J medium (Table 1). Therefore, in our assay, specimen volume could not be the determined factor leading to the significant difference between those different culture methods.

Budgetary constraint is a major consideration affecting the choice of diagnostics in developing countries. In China, the prices of vials for Myco/F lytic assay and for MGIT960 culture are similar, whereas BD Bactec 9120 system is approximately half the price of MGIT 960 system. Additionally, since Myco/F lytic assay is less labor needed, so it should be more cost-effective than MGIT960 culture.

## Conclusion

Both Myco/F lytic and MGIT960 system were superior to L-J medium for recovery of MTC from sterile body fluids. The Myco/F lytic system culture is very labor-saving, can be considered as a promising alternative to MGIT system for diagnosing EPTB.

## Material and Methods

### Ethical Statement

The ethical approvals for this study were obtained from Beijing Chest Hospital Ethics Committee. A written informed consent was acquired from each participant. The present study was conducted inaccordance with the Declaration of Helsinki Principles.

### Sample collection

The study was conducted from April to November 2015 at Beijing Chest Hospital (Beijing, China), which is the only national referral TB center in China. A total of 214 sterile body fluids, including 114 pleural fluid and 100 pus, were collected from 214 patients with a clinical diagnosis of EPTB.

TB was diagnosed on the basis of a composite gold standard including clinical characteristics, microbiological, histopathological, cytological, and radiological examinations, and response to anti-tubercular therapy. A case was considered clinically diagnosed EPTB, if mycobacterial evidence or any two of the other criteria mentioned in the composite gold standard were met.

### Sample processing

Four milliliters of sterile body fluid was inoculated at bedside into a Myco/F lytic culture vial and incubated in the Bactec 9120 system (Becton, Dickinson and Company, USA) within two hours. Another 50 ml pleural fluid or 5 ml pus was collected in a sterile Falcon tube, transported to the laboratory, and spun at 3,000 × g, and the pellet was resuspended with 800 μl phosphate buffer. Then 500 μl suspensionswere inoculated into a 7-ml MGIT tube and incubated in the MGIT 960 system (Becton, Dickinson and Company, USA), while 100 μl suspensions were inoculated onto L-J medium (Encode Medical Engineering Co., Ltd, China). The L-J media were incubated at 37 °C in aerobic atmosphere. The suspensions were also stained with hot Ziehl-Neelsen (ZN) staining before examination and grading for acid-fast bacilli (AFB) following the protocol recommended by Chinese anti-TB Association^20^.

When a culture flagged positive by Myco/F lytic or MGIT 960 system, the time to positivity (TTP) was recorded. L-J tubes were incubated at 37 °C and examined weekly for growth for a maximum of 8 weeks. All positive liquid cultures were confirmed by ZN staining. For all the culture positive tubes, Capilia TB-Neo (Tauns Laboratories, Numazu, Japan^21^) was performed to differentiate MTC from non-tuberculous mycobacteria. Bacterial contamination was ruled out by incubating the sample on a blood agar plate.

### Statistical analysis

The recovery rates and contamination rates of different methods were compared using χ^2^ test. The *t* distribution was used to determine the statistical significance of the times to positivity. Statistical analyses were performed with SPSS (version 19.0). The differences were considered significant when P < 0.05.

## Additional Information

**How to cite this article**: Wang, G. *et al.* Evaluation of the efficacy of Myco/F lytic system, MGIT960 system and Lowenstein-Jensen medium for recovery of *Mycobacterium tuberculosis* from sterile body fluids. *Sci. Rep.* **6**, 37757; doi: 10.1038/srep37757 (2016).

**Publisher's note:** Springer Nature remains neutral with regard to jurisdictional claims in published maps and institutional affiliations.

## Figures and Tables

**Table 1:** Recovery rates of *Mycobacterium tuberculosis* complex in each culture system and in combinations of each two systems.

Sample type	No. (%) of isolates recovered									
	Total, all media	Myco/F lytic + MGIT	Myco/F lytic + L-J	MGIT + L-J	Myco/F lytic	MGIT	L-J	Recovered by Myco/F lytic only	Recovered by MGIT only	Recovered by L-J only
Pleural fluid	36 (100)	35 (97.22)	35 (97.22)	28 (77.78)	32 (88.89)	25 (69.44)	13 (36.11)	9 (25.00)	2 (5.56)	1 (2.78)
Pus	67 (100)	66 (98.51)	62 (92.54)	57 (85.07)	57 (85.07)	53 (79.10)	31 (46.27)	10 (14.93)	4 (5.97)	1 (1.49)
Total	103 (100)	101 (98.06)	97 (94.17)	85 (82.52)#	89 (86.41)	78 (75.73)	44 (42.72)#	19 (18.45)	6 (5.83)	2 (1.94)

MGIT, BACTEC MGIT 960 system; L-J, Lowenstein-Jensen medium.

#, χ^2^ test for differences in recovery rates: total Myco/F lytic versus total L-J, P < 0.0001; total MGIT 960 versus total L-J, P < 0.0001;

Myco/F lytic + MGIT 960 versus MGIT 960 + L-J, P < 0.0001; Myco/F lytic + L-J versus MGIT + L-J, P = 0.009; Myco/F lytic + MGIT 960 and Myco/F lytic + L-J, P = 0.149.

**Table 2:** Smear and culture results for 103 positive cultures.

Smear result	No. (%) of isolates recovered			
	No. of smears	Myco/F lytic	MGIT	L-J medium
Negative	69	64 (92.75)	54 (78.76)	34 (49.28)
Positive	34	24 (70.59)	23 (67.65)	9 (26.47)
1+	18	13 (72.22)	11 (61.11)	5 (27.78)
2+	12	8 (66.67)	10 (83.33)	3 (25.00)
3+	2	1 (50.00)	0 (0)	0 (0)
4+	2	2 (100.00)	2 (100.00)	1 (50.00)
Total	103	88 (85.44)	77 (74.76)	43 (41.75)

MGIT, BACTEC MGIT 960 system; L-J, Lowenstein-Jensen medium.

#, χ^2^ test for differences in recovery rates: for smear negative specimens Myco/F lytic versus MGIT 960, P = 0.016; Myco/F lytic versus L-J, P < 0.0001; MGIT 960 versus L-J, P < 0.0001. For smear positive specimens Myco/F lytic versus MGIT 960, P = 0.793; Myco/F lytic versus L-J, P < 0.0001; MGIT 960 versus L-J, P = 0.0001.
